# Pneumococcal pneumonia on the job: Uncovering the past story of occupational exposure to metal fumes and dust

**DOI:** 10.1002/ajim.23352

**Published:** 2022-03-29

**Authors:** Kjell Torén, Rajen N. Naidoo, Paul D. Blanc

**Affiliations:** ^1^ Occupational and Environmental Medicine, School of Public Health and Community Medicine, Institute of Medicine, Sahlgrenska Academy University of Gothenburg Gothenburg Sweden; ^2^ Department of Occupational and Environmental Medicine Sahlgrenska University Hospital Gothenburg Sweden; ^3^ Discipline of Occupational and Environmental Health University of KwaZulu‐Natal Durban South Africa; ^4^ Department of Medicine, Division of Occupational and Environmental Medicine University of California San Francisco California USA

**Keywords:** history of medicine, industrial disease, manganese, metal fume, occupational health, welding

## Abstract

The objectives of this study are to elucidate the early history of risk for pneumococcal pneumonia from occupational exposure to metal fumes and dusts, and to demonstrate the importance of searching older literature when performing reviews. We performed manual searching for articles in the Library of the Surgeon General's Office (the precursor to Index Medicus), in the Hathi Trust database, in PubMed, andby screening reference lists in literature appearing before the introduction of PubMed. An early body of literature, from the 1890s onward, recognized that pneumonia was linked to “Thomas slag,” a steel industry byproduct containing iron, manganese, and lime. Researchers, mainly in Germany, showed that workers in metal‐dust‐exposed occupations, especially using manganese, manifested an increased incidence of pneumococcal pneumonia. An outbreak of pneumococcal pneumonia in the 1930s implicated manganese fume in its etiology. In the immediate post‐World War II period, there was a brief flurry of interest in pneumonia from exposure to potassium permanganate that was soon dismissed as a chemical pneumonitis. After a hiatus of two decades, epidemiologic investigations drew attention to the pneumonia risks of welding and related metal fume exposure, bringing renewed interest to the forgotten role of pneumococcal pneumonia as an occupational disease. Occupational or environmental inhalation of manganese, iron, or irritants may be causally related to increased pneumococcal pneumonia risk. In particular, the risk associated with manganese seems to be overlooked in recent literature. An important conclusion is the importance of obtaining additional evidence through a deeper assessment of the literature in a broad historical context.

## 1 INTRODUCTION

The stuttering history of describing pneumonia contracted on‐the‐job illustrates how our understanding of disease can be retarded by a form of cyclical amnesia that frequently plagues both the research and the practice of occupational pulmonary medicine. Indeed, the observation that occupational exposure to metal dust and fume is strongly linked to pneumonia has been made and then forgotten more than once. During the period from1890 to 1960 a number of studies relevant to this question were published, but each burst of attention was followed by a hiatus in research. In 1994, a seminal paper brought new and sustained attention to this topic.^1^ However, neither that publication nor those that came after sufficiently emphasized the earlier, rich literature on this topic.^2^, ^3^, ^4^, ^5^, ^6^

The aim of this in‐depth historical review is to show that occupational exposure to metals and fumes and dusts has been strongly and repeatedly linked to pneumococcal pneumonia in multiple reports. Nonetheless this evidence has not been considered in an integrated manner, largely because each time the observation has been made, the previous biomedical literature has had to be rediscovered, leading to unnecessary fragmentation and confusing nosology.

The modern medical history of pneumonia is characterized by disease nosology that changed with the insights gained from evolving microbiologic and radiographic techniques. In 1880, Pasteur in France and Sternberg in the United States performed the first isolation of the pneumococcus.^7^, ^8^ By the middle of 1880s, it became finally clear that most acute pneumonia was bacterial and that infection with *Streptococcus pneumoniae* was a main cause of such bacterial pneumonia.^9^ The earlier term “croupous pneumonia” for severe illness later gave way to “lobar pneumonia” when it was possible to radiographically assess patients.

## 2 EARLY OCCUPATIONAL PNEUMONIAS

The potential role of occupational factors in acute bacterial pneumonia initially received scant consideration. Nonetheless, a relatively early paper, a 1900 review of the public health aspects of pneumonia, was published by the prominent British physician Newsholm.^10^ Addressing the “influence of occupation,” he called attention to the Registrar‐General's data for deaths by occupation for 1890–1892 among those aged 25–65 years. Newsholm noted that the overall death rate for pneumonia was 107 per 1000 and was as low as 45 per 1000 among the clergy, whereas among iron and steel manufacturers and among coal‐heavers, the rate was 248 and 249, respectively.

### 2.1 Thomas slag pneumonia

Even before 1900, there was recognition that acute pneumonia might be linked to a byproduct of steel manufacturing, a now obscure material then known as “Thomas slag.” In 1878, the British cousins Sidney Gilchrist Thomas and Percy Carlyle Gilchrist modified the Bessemer process for steel production, adding limestone to the process. Their patented innovation involved covering the interior area of the Bessemer converters with a mixture of magnesia, dolomite, tar, and lime. This resulted in an advantageous extraction of phosphorous, an unwanted contaminant, from the iron being converted to steel. The new process generated large amounts of alkaline slag, eponymously named Thomas slag (in German, *Thomasschlacken*). It soon was recognized that this otherwise worthless slag had monetary value as a fertilizer feedstock due to its high phosphorous content. To exploit this, the slag was crushed in special mills into a very fine powder, as its efficiency as a fertilizer depended on the minuteness of the powder. This process, as well as the transportation and emptying of the sacks of ground slag, generated very high levels of dust, very likely to be in the respirable range.

By the late 1880s, in Germany, France, and England, where Thomas slag processing had become a prominent industrial process, it became abundantly evident that Thomas slag workers suffered from a high prevalence of acute, frequently fatal, pneumonia. Dosenheimer^11^ was the first to report the phenomenon in Germany. In Nantes, France, an epidemic of pneumonia was reported among workers processing Thomas slag: a commission concluded that dust from the milling of the slag was an important cause of the pneumonia.^12^ Nothing was mentioned about microorganisms, but the commission's report initiated a debate as to whether the epidemic had been caused by contamination (contagion) from other workers. In May 1888 in Middlesbrough, Yorkshire, England, a drastic increase of deaths from a severe acute pleuro‐pneumonia was reported.^13^ Deaths typically occurred on the third to the fifth day of illness. The workmen themselves attributed it to inhalation of dust from a newly opened Thomas slag processing facility. An investigation initiated by the local government concluded that exposure to “slag dust” was not the primary source of illness but allowed that is might be an “assisting cause” making the exposed workers more susceptible to disease. A subsequent report from Middlesbrough concluded that the extremely fine dust created a distinct predisposition to pneumonia and that when the pneumonia did occur, the fatality rate was very high.^14^

Three ensuing publications from Germany described additional outbreaks of severe croupous pneumonia with high mortality among groups of workers employed in extremely dusty Thomas slag mills.^15^, ^16^, ^17^ The conclusion from these reports was that the dust irritated the lungs, giving pneumococci favorable conditions to grow. As importantly, the composition of the slag was characterized in two of these reports as containing phosphate, lime, silica, iron oxides, and manganese compounds. By 1895, comparative data from Germany showed that the multiyear cumulative prevalence of pneumonia was 14.4% (mortality 6.5%) among the Thomas slag millers compared with 2.0% (mortality 0.7%) among steel mill workers.^18^ That study considered the lime in the slag as the irritative causal factor. By the turn of the 20th century, Thomas slag had crossed the Atlantic; a 1903 report by the United States Bureau of Labor reported that the dust arising from grinding basic Thomas slag caused severe respiratory disease.^19^

Thomas slag pneumonia became a widely enough recognized phenomenon to be covered in relevant textbooks. As early as 1896, for example, a German occupational medicine specialty textbook noted that work in Thomas slag mills was associated with lethal pneumonias.^20^ The stated opinion was that the alkaline lime eroded the mucous membrane of the airways and injured the deeper tissue; there was no mention of pneumococci in that text. In the first International Labour Organization Encyclopedia of 1930, the definitive indicator of widespread recognition of the phenomenon in the discipline of occupational health, an extensive chapter was dedicated to the topic, concluding that exposure to Thomas slag caused pneumococcal pneumonia.^21^ Also, on the international level, at the eighth International Congress for Occupational Accidents and Occupational Medicine held in Frankfurt am Main, 1938, one of the keynote lectures acknowledged that the milling of Thomas slag increased the risk for bacterial pneumonia and that the irritating properties of the slag promoted bacterial growth.^22^

In the years bridging the First World War into the Second World War, the German occupational medicine literature continued to give considerable attention to work‐related pneumonia in the *Thomasschlacken* trade.^23^, ^24^ Underscoring this, in 1926, *Thomasschlacken* pneumonia was officially listed in Germany as an occupational disease.^25^ Throughout the 1930s and even into the war years, German researchers continued to describe the epidemiology of very severe pneumonia, with high mortality, among workers processing Thomas slag, with exposure to manganese specifically considered as a possible etiologic factor, in addition to irritating dust.^26^, ^27^ Ultimately, German researchers could not confirm experimentally that manganese oxide, the form of manganese in Thomas slag, was the etiologic factor in *Thomasschlackenpneumonie*.^28^ Researchers returned to the earlier view that the adverse effects of exposure to Thomas slag most likely were mediated through a local irritating effect of this very alkaline material. But in any event, in the post‐War period Thomas slag pneumonia became irrelevant, as the use of Thomas slag as fertilizer decreased sharply as chemical nitrogen, phosphorous, and potassium fertilizers came into broad use.^29^ The Thomas slag industry disappeared and, in modern occupational medicine textbooks, the phenomenon of Thomas slag pneumonia is no longer mentioned at all.

### 2.2 The Norwegian story

In the meantime, another form of fume‐associated pneumonia had emerged, an instance in which exposure to manganese was highly suspected as an etiologic factor. In 1923, a manganese smelter was established in the Norwegian town of Sauda, located in a narrow valley in southwestern Norway. The new facility emitted large amounts of manganese‐rich smoke and, as the valley was very narrow, most of the households in the village were heavily exposed to ambient pollution from this source.

In the late autumn of 1923, local physicians noted an increased occurrence of lobar pneumonia (croupous pneumonia) with all the classic clinical signs, including the abrupt onset of high fever, dyspnea, cyanosis, and radiological signs of lobar pneumonia. During the years that immediately followed, physicians in Sauda continued to note a considerably increased occurrence of fatal pneumonia in the local population. Initial investigations concluded, incorrectly, that this accumulation of lethal cases of pneumonia was not associated with the pollution from the smelter.^30^ In the decade that followed, the mortality and morbidity from lobar pneumonia in Sauda continued to be high compared with the rest of Norway. In 1938, a local physician in Sauda, Dagfinn Elstad, presented a study in which he showed quite convincingly that the epidemic probably was linked to emission of manganese oxides from the smelter.^31^ In that report, he presented data documenting that the mortality in pneumonia was much higher in Sauda compared with the general Norwegian population, showing that the age‐adjusted mortality rate from croupous pneumonia was around 40/10,000 in Sauda compared with the national rates of 4/10,000. The ferromanganese and silicomanganese produced at the Sauda smelter contained 80%–90% manganese and 14% iron, but the ferrosilica contained only 25%–30% manganese and 60%–70% iron. Elstad's data further showed that the mortality and morbidity in Sauda from croupous (lobar) pneumonia were closely related to the production of ferromanganese: the years with high production of ferromanganese saw higher mortality as compared with lower mortality in years when the production of ferrosilica was higher.

At the eighth International Congress for Occupational Accidents and Occupational Medicine in Frankfurt am Main in September 26–30, 1938, on Wednesday September 29, 1938 (the day of the München agreement), the third presentation was a study by Elstad, “Beobachtungen über Manganpneumonien.”^32^ Dagfinn Elstad was acknowledged in the international medical press and he was cited by the leading researchers in the field.^33^

### 2.3 Manganese or iron?

German researchers also raised the possibility that it was manganese that acted as an important factor in work‐related pneumonia observed not only in Thomas mills but also in other industries.^34^, ^35^, ^36^ This hypothesis received support from reports of occupationally associated pneumonia from other manganese exposure scenarios (Table 1). In manganese mines and manganese smelters, multiple reports described severe pneumonia in workers with heavy exposure to manganese dioxide.^23^, ^24^, ^36^, ^38^, ^39^, ^41^, ^42^, ^47^ In addition, harbor workers loading manganese ore were noted to have an increased incidence of pneumonia, documented in two nearly simultaneous reports half a world apart.^38^, ^47^ In another study, Baader,^48^ in addition to emphasizing the neurotoxicological manifestations of manganese among German battery factory workers, also noted an increased occurrence of severe pneumonias from this metal. In subsequent reviews of the topic, Büttner^49^, ^50^ described the occupational manganese exposure in Germany in 14 mines, 6 ferromanganese mills, and 55 battery factories. He concluded that manganese‐exposed workers had considerably higher mortality due to pneumonia compared with other miners in the Ruhr area. As a preventive action, he argued for dust reduction in these workplaces. German researchers also visited manganese mines in Egypt, where they described a high mortality from pneumonia.^46^, ^51^ Two German doctoral theses about occupational manganese exposure and pneumonia were submitted in 1938.^44^, ^45^ In contrast to the attention given by German researchers to pneumonia as a potential effect of occupational exposure to manganese or iron, in the United Kingdom and the United States, this topic received scant notice. A brief summary of Baader's work on manganese that appeared in the *Journal of the American Medical Association*, for example, focused on neurological disease, although it did include a single sentence on pneumonia.^52^

**Table 1:** Selected biomedical publications on pneumonia in workers in manganese mines, manganese smelters, and battery factories, 1921–1939

Year, author, and reference	Country	Publication language	Occupational environment and exposures	Outcomes
1921, Brezina^23^	Germany	German	Processing of manganese ore (Braunsteinmühle) 1911–1913	50% of the workers died due to severe pneumonia
1921, Brezina^24^	Germany	German	Manganese mines	Increased pneumonia mortality
1927, Registrar General^37^	UK	English	Work in brass foundries and work as furnacemen, puddlers, and metalmolders	Increased standardized mortality from pneumonia. Zinc fumes were discussed as a possible cause.
1930, Schopper^36^	Germany	German	Postmortem analysis of two miners	Died from pneumonia. The lungs contained foreign material rich in manganese
1932, Bubarev^38^	Georgia (Russia)	Russian	Stevedores loading and unloading manganese ore	37 Out of 70 workers had severe pneumonia
1932, Freise^39^	Brazil	German	Stevedores in Rio de Janeiro handling manganese ore and manganese miners in Minas Gerais. The ore contained 50% MnO_2_	Of 442 workers, 61% have had pneumonia
1932, Brundage et al.^40^	USA	English	Steel mills in Pennsylvania	High pneumonia mortality in the blast furnace department, coke ovens, and open heart departments
1933, Bickert^41^	Germany	German	Two plants processing manganese ore, altogether 337 workers were employed 1920–1931	Seven workers had croupous pneumonia and 22 had manganism
1935, Gallego^42^	Spain	Spanish	Manganese miners in Andalusia	Six cases of pneumonia among 64 miners
1937, Vigliani^47^	Italy	Italian	Processing of manganese ore rich in MnO_2_	After 10 days employment, the worker died in a pneumococcal pneumonia
1938, Büttner and Lenz^43^	Germany	German	Miners in the second largest manganese mine	Mortality due to pneumonia 1926–1936 Manganese miners: 58% Iron‐ore miners: 20% Manual workers: 10%
1938, Gundel and Heine^34^	Germany	German	A large smelter producing ferrochrome, ferrosilicium, ferrowolfram, ferromolybden, and ferromanganese	Pneumonia mortality; Exposed workers: 1.2%–1.6%; Miners: 0.1%
1938, Bauer^44^	Germany	German	PhD thesis with review of the literature, description of 13 battery factories and four manganese ore processing plants. 104 Workers were classified as exposed to manganese	1928–1937: 14 Cases of pneumonia, and of these 11 died (79%) from pneumonia In 1934, the pneumonia mortality was 7.9/10,000 persons
Wenig, 1938^45^	Germany	German	PhD thesis with review of the literature	Postmortem analysis of five cases of pneumonia among manganese‐exposed workers
1939, Baader^46^	Egypt	German	Manganese miners in Sinai. The ore contained 40%–60% MnO_2_	In 10 years, there were 99 cases of pneumonia with 42% mortality

In this period, exposure to iron also was considered as an etiologic factor for pneumonia of potential importance. Data from a manganese mine at Giessener in the Rhineland demonstrated high mortality from croupous pneumonia compared with manual workers and iron‐ore miners.^43^ The mine ore contained 17% manganese but also 20% iron. The iron‐ore miners without manganese co‐exposure also manifested increased pneumonia mortality and the authors discussed whether iron could be a pathogenic factor, in addition to manganese. Moreover, in both the United Kingdom and the United States, there were early reports of an increased occurrence of pneumonia among steel mill and other metal workers, potentially implicating iron,^37^, ^40^ as well as the early study by Newsholm.^10^

### 2.4 Pneumonia or chemical pneumonitis?

Shortly after World War II, it was not in metal manufacturing, but rather in the pharmaceuticals industry, that British attention was drawn to manganese as a possible risk factor for pneumonia. In 1946, a British researcher reported a clear‐cut cluster of pneumonia in a factory producing potassium permanganate.^53^ The clinical picture was that of a febrile lobar pneumonia, but also of severe respiratory irritation with bronchitis, pharyngitis, and epistaxis. The outbreak occurred in a manufacturing facility where manganese dioxide was ground, reacted with potassium hydroxide and lime, roasted in rotary kilns, and then purified through electrolysis. The electrolytic process was extremely dusty, leading to heavy exposure to manganese dioxide and lime in the workforce, with dust levels up to 40 mg/m^3^ reported. For the period 1938–1945, the incidence of pneumonia was 26/1000 among the exposed as compared with 0.7/1000 among unexposed male referent workers.

These observations, suggesting that infectious pneumonia was indeed an outcome of manganese exposure, were counterbalanced by animal experimentation data included in the same publication. The animal component of the study found that mice exposed to manganese dust from the milling room and control mice exposed to lime dust (calcium carbonate) both showed marked manganese‐associated pulmonary inflammation. Moreover, in mice further exposed to pneumococci, the mortality was similar in both the manganese‐exposed and lime‐dust‐exposed control animals. The authors concluded that workers were at increased risk of pneumonia, but that this might be wholly an irritant phenomenon without increased susceptibility to pneumococcal infection. A follow‐up study reported four additional workers suffering from pneumonia but also presented additional experimental animal data, the latter showing lung injury from intratracheal installation of manganese oxide. On balance, the authors concluded that the syndrome was a “pneumonitis,, not primary a bacterial pneumonia.^54^

These two publications, both by Lloyd Davis, are particularly important in the history of occupational exposure, especially to manganese and the associated risk of pneumonia. These findings essentially reinforced previous German research showing that workers in manganese‐exposed occupations had an increased occurrence of severe pneumonia. Further, also like the German studies, the potassium permanganate papers concluded that exposure to irritants were playing a mechanistic role. Nonetheless, reconceptualizing the condition as a pneumonitis rather than pneumonia downplayed an infectious etiology. A critical reassessment of the human data in these studies, however, suggests that the construct of a toxic pneumonitis applied to these observations is far from convincing, given that the reported clinical picture resembles severe infectious pneumonia, not an irritant pneumonitis. As these two studies were published in the English language at the advent of a new post‐World War II period in industrial medicine, their impact likely was disproportionate to the far larger body of German language biomedical literature that preceded them.

In the decades that followed the Lloyd Davies studies, the potential link between occupational exposures and pneumococcal pneumonia was uncommonly considered, although the topic did not fall into the complete obscurity that Thomas slag pneumonia had. In the mid‐1950s, for example, manganese was alluded to as a risk factor for lobar pneumonia in major English and German language textbooks of occupational medicine.^55^, ^56^ Moreover, occupational data from mines and smelters did sporadically appear.^57^, ^58^ Ultimately though, it would not be mining or smelting but another industrial process, welding, which would drive renewed interest in the potential industrial causes of pneumonia.

## 3 WELDING

The process we recognize today as welding first emerged in 1881 when a Dutchman, Auguste de Méritens, reported the use of arc heat to join metal plates. Russian technologists further refined the process, especially by developing a metal electrode that itself melted during welding, with the molten metal transferred to the weld. Oscar Kjellberg, a technologist in Gothenburg, Sweden, in 1906, introduced electric arc welding by inventing the covered electrode, a method which still is used today in most welding processes. In 1920, Kjellberg applied his process to construct the first all‐welded ship, the SS Esab.^59^ Welding on mild and low‐alloy steels (metals with relatively low manganese content) accounts for ~90% of all welding. Thus, the main component of all welding fume generated by the base metal being welded is iron oxide (Fe_3_O_4_). However, because of variability in welding rod components, there are also a variety of different manganese oxides present in welding fumes, mainly Mn_2_O_3_ and MnO (Mn^3+^ and Mn^2+^).^60^

An early study on welders at four Kaiser shipyards in the United States did not show an increased risk of pneumonia, but the comparison groups were not representative, making the findings difficult to interpret.^61^ In contrast, two US mortality studies found an increased pneumonia mortality among welders.^62^, ^63^ The occupational association with pneumonia was also highlighted by the Registrar General in England and Wales, noting that welders, cutters, and braziers had a striking excess of pneumonia.^64^ It was not until the Registrar General's Supplement for 1970–1972, however, that it first was suggested that the excess of pneumonia might specifically be due to welding fumes.^65^ Another study of pneumonia mortality in British naval shipyard welders was inconclusive, as there were no observed cases of either bronchopneumonia or lobar pneumonia.^66^ A subsequent study of British shipyard welders, however, did observe increased infectious pneumonia mortality.^67^

It was Coggon et al.^1^ who spearheaded the further investigation that was needed. This investigation extended analysis of UK national occupational mortality from pneumonia 1959–1963 and 1970–1972, based on additional information from the Registrar General in England and Wales. This landmark analysis identified four occupational groups with increased mortality from pneumococcal and unspecified lobar pneumonia (International classification of diseases 9, 481): welders (Standardized Mortality Ratio [SMR] 255, 95% confidence interval [CI]: 192–332); molders and coremakers (SMR 292, 95% CI: 173–461); annealers, hardeners, and temperers (SMR 392, 95% CI: 107–1003); and sheet‐metal workers (SMR 190, 95% CI: 117–290). There was no observed increased risk of bronchopneumonia, as opposed to lobar, among any of these occupations. The authors discussed metal fumes as a possible explanatory factor and exposure to iron specifically was invoked as the responsible agent. An editorial appearing in the same issue of *Lancet* and authored by Kennedy,^68^ a leading occupational hygienist, concluded that this was new knowledge, suggesting that lobar pneumonia should be classified as an occupational lung disease. She went further though, raising the insightful point that this might not be limited solely to welders.^68^ Nonetheless, manganese, an important constituent of most welding fumes, went unmentioned and none of the relevant previous literature was cited either in the Coggon publication or its accompanying editorial.^1^, ^68^

The 1994 Coggon publication was followed be a series of elegant epidemiological investigations of the association between metal fume and pneumonia. These studies were included in a 2019 systematic review that carried out a pooled analysis of seven studies of pneumonia in welders or in individuals with metal fume exposure, finding that the median attributable fraction of disease linked to exposure was 52.5%.^69^ Since that review, additional epidemiologic data further support the association between metal fumes and pneumococcal pneumonia.^70^, ^71^ Although these modern epidemiological observations are powerful, they do not directly address specific questions of mechanism. Experimental data, however, also are germane to this question. Inhalation of metals, in particular manganese and iron, and other inorganic dusts and fumes may increase infection risk, for example, by impairing pulmonary clearance of pathogens.^72^

## 4 CONCLUDING REMARKS: THE CRITICAL ROLE OF AN HISTORICAL CONTEXT

One conclusion that clearly can be drawn from this historical review is that the occupational or ambient environmental inhalation of manganese, iron, or possibly a caustic irritant such as lime, indeed is likely to be causally related to increased pneumococcal pneumonia risk. This is strongly supported through in‐depth assessment of the biomedical evidence in its broad historical context. Were we to have considered any one of the exposure scenarios isolated in time and space, for example Thomas slag or welding fume alone, the conclusion would be far weaker.

An equally important conclusion that might be drawn from this review is that the full historical record can be minimized, fragmented, or overlooked altogether in reviews and original papers of a topic such as this. The barriers to broader historical consideration likely are multiple and generalizable beyond the question of metal fume and pneumonia that are the focus of this review. Modern reviews typically rely on standard electronic databases that do not reach back sufficiently enough in time to capture the historical scope of many topics. This limitation can be compounded further by restricting references to English language publications only, which in our case would have omitted most of the core Thomas slag literature, as well as the key references for the Norwegian manganese smelter pneumonia outbreak. To overcome these barriers to increased historical consideration in interrogating relevant topics, we would strongly advise authors to complement PubMed with searches in the Index catalog of the Library of the Surgeon General's Office (1880–1961), which is the precursor to Index Medicus. This includes a wide range of languages and is also available for online searching, https://www.nlm.nih.gov/pubs/techbull/mj04/mj04_cat.html. Another useful resource is the Hathi Trust database, https://www.hathitrust.org, the source for several of our citations.

Finally, and perhaps most critically, unless the historical record is given attention in the first place, the connection to past experience will never be made. This is very much in line with the clinical centrality of taking an occupational history in a patient who may have a work‐related disease.

Our review shows that repeated cycles of discovery and forgetfulness characterize the topic of metal fume as an occupational and environmental etiologic factor in pneumococcal pneumonia. In‐depth studies of other exposures support the view that such cycles characterize additional exposures as well, for example, the histories of manganese neurotoxicity,^60^ chlorine inhalation injury,^73^, ^74^ and carbon disulfide toxicity.^75^ Although this phenomenon also is likely to extend to other topics in occupational and environmental medicine, further analysis on a case‐by‐case basis is warranted to establish this as a generalizable pattern. To that end, we strongly would encourage other investigators to take an integrated, historically well‐informed approach when considering relevant questions of occupational etiology and causation.

## CONFLICTS OF INTEREST

The authors declare no conflicts of interest.

## DISCLOSURE BY AJIM EDITOR OF RECORD

John Meyer declares that he has no conflict of interest in the review and publication decision regarding this article.

## AUTHOR CONTRIBUTIONS

Kjell Torén designed the study, performed the literature searches, and wrote the first draft of the manuscript. All authors have full access to the data, interpreted the data, and assisted in the drafting of the manuscript. Kjell Torén had the final responsibility to submit the manuscript.

## ETHICS STATEMENT

Not applicable, as the study solely is based on published material
